# Supplementary material for: Hybrid Complex Coacervate
Source: Polymers (Basel). 2020 Feb 4;12(2):320. doi: 10.3390/polym12020320 (PMC7077495; doi:10.3390/polym12020320)
Supplement: Supplementary file 1 [file polymers-12-00320-s001.pdf]

polymers

Supporting Information

Hybrid Complex Coacervate

Marco Dompè [1], Francisco Javier Cedano-Serrano [2], Mehdi Vahdati[2], Dominique Hourdet[2], Jasper van der Gucht[1], Marleen Kamperman[1,3] and Thomas E. Kodger[1,*]

[1] Laboratory of Physical Chemistry and Soft Matter, Wageningen University & Research, Stippeneng 4, 6708 WE Wageningen, The Netherlands; marco.dompe@wur.nl (M.D.); jasper.vandergucht@wur.nl (J.v.d.G.); marleen.kamperman@rug.nl (M.K.); thomas.kodger@wur.nl (T.K.)
[2] Soft Matter Sciences and Engineering, ESPCI Paris, PSL University, Sorbonne University, CNRS, F-75005, Rue Vauquelin 10, Paris, France; francisco.cedano@espci.fr (F.J.C.S.); mehdi.vahdati@espci.fr (M.V.); dominique.hourdet@espci.fr (D.H.)
[3] Laboratory of Polymer Science, Zernike Institute for Advanced Materials, University of Groningen, Nijenborgh 4, 9747 AG Groningen, The Netherlands; marleen.kamperman@rug.nl (M.K.)
* Correspondence: thomas.kodger@wur.nl;

Keywords: complex coacervation, nanofillers, nanocomposites, polyelectrolytes, underwater adhesion, poly(N-isopropylacrylamide)

1. ^{1}H-NMR spectra of graft copolymers

PAA-*g*-PNIPAM (Figure S1): PAA (^{1}H-NMR, 400 MHz, D_2O, δ (ppm)): 1.45-1.71 (2H,CH_2 backbone), 2.13 (1H, CH backbone). PNIPAM (^{1}H-NMR, 400 MHz, D_2O, δ (ppm)): 1.15 (6H, CH_3), 1.59 (2H, CH_2 backbone), 2.02 (1H, CH backbone), 3.90 (1H, CH).

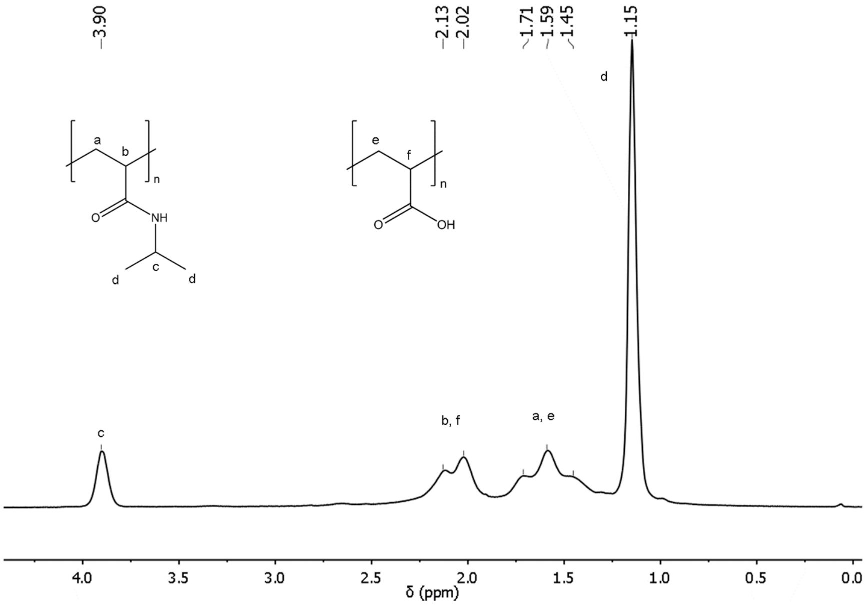

Figure S1 ^{1}H-NMR spectrum of PAA-*g*-PNIPAM

The molar ratio of PNIPAM sidechains was determined as follows. At first, the area of the peak at 3.90 ppm was set to 1.0. Afterwards, in order to get the PAA contribution to the ^{1}H-NMR spectrum, the area between 1.25 ppm and 2.5 ppm was subtracted by 3.0 (number of hydrogens belonging to the PNIPAM backbone) and successively divided by 3.0 (number of hydrogens belonging to the PAA backbone). The molar ratio of PNIPAM sidechains was then obtained by dividing the area relative to

one PNIPAM hydrogen (1.0) by the sum of the areas relative to one PAA hydrogen and one PNIPAM hydrogen.

PDMAPAA-*g*-PNIPAM (Figure S2): PDMAPAA (^{1}H-NMR, 400 MHz, D_2O, δ (ppm)): 1.60 (1H, CH backbone), 1.96 (2H, CH_2), 2.05 (1H, CH backbone), 2.90 (6H, CH_3), 3.15 (2H, CH_2), 3.23 (2H, CH_2). PNIPAM (^{1}H-NMR, 400 MHz, D_2O, δ (ppm)): 1.15 (6H, CH_3), 1.73 (2H, CH_2 backbone), 2.20 (1H, CH backbone), 3.90 (1H, CH).

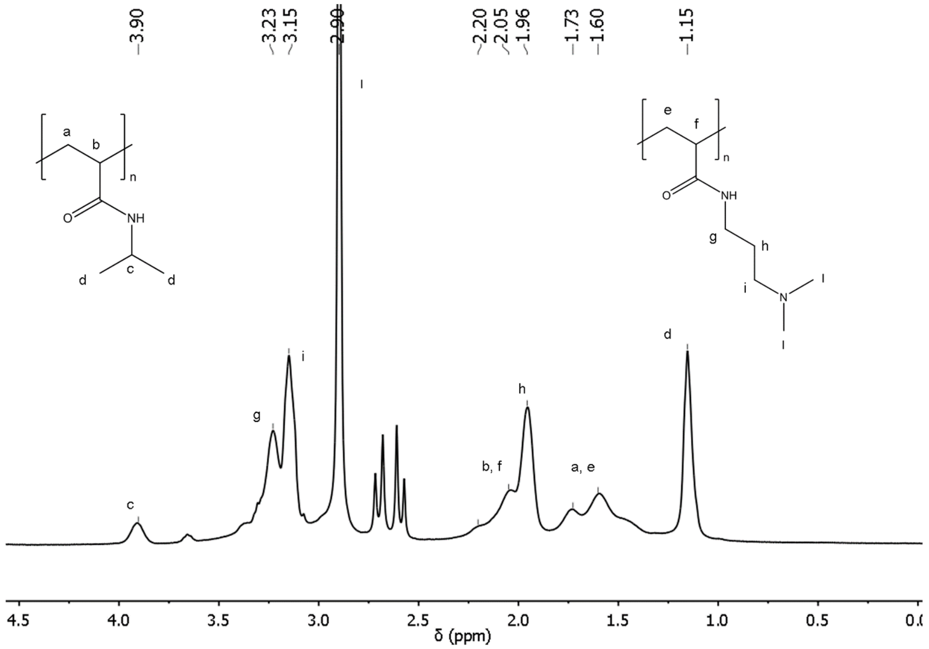

Figure S2 ^{1}H-NMR spectrum of PDMAPAA-*g*-PNIPAM

The mol% of PNIPAM sidechains was determined as follows. At first, the area of the peak at 1.15 ppm, corresponding to 6 hydrogens in the PNIPAM isopropyl group, was set to 1.0. Afterwards, the area of the peak at 2.90, corresponding to 6 hydrogens in the PDMAPAA dimethylamine group, was determined. The molar ratio of PNIPAM sidechains was then obtained by calculating the ratio between the PNIPAM signal (1.0) and the sum of the PNIPAM and PDMAPAA signals. The signal present around 2.6 is due to the presence of citrate counterions.
